# Information-Theoretic Inference of Large Transcriptional Regulatory Networks

**DOI:** 10.1155/2007/79879

**Published:** 2007-06-24

**Authors:** Patrick E Meyer, Kevin Kontos, Frederic Lafitte, Gianluca Bontempi

**Affiliations:** 1 ULB Machine Learning Group, Computer Science Department, Université Libre de Bruxelles, Brussels 1050, Belgium

## Abstract

The paper presents MRNET, an original method for inferring genetic networks from microarray data. The method is based on maximum relevance/minimum redundancy (MRMR), an effective information-theoretic technique for feature selection in supervised learning. The MRMR principle consists in selecting among the least redundant variables the ones that have the highest mutual information with the target. MRNET extends this feature selection principle to networks in order to infer gene-dependence relationships from microarray data. The paper assesses MRNET by benchmarking it against RELNET, CLR, and ARACNE, three state-of-the-art information-theoretic methods for large (up to several thousands of genes) network inference. Experimental results on thirty synthetically generated microarray datasets show that MRNET is competitive with these methods.

## 

[1,2,3,4,5,6,7,8,9,10,11,12,13,14,15,16,17,18,19,20,21,22,23,24,25,26,27,28]
